# Factors associated with completion of 6 months of isoniazid preventive therapy among under-five children exposed to tuberculosis patients in Blantyre, Malawi

**DOI:** 10.4102/jphia.v17i1.1528

**Published:** 2026-03-13

**Authors:** Glory T. Mzama, Kruger Kaswaswa, Tobias Chirwa, Juliana Kagura, Latifat Ibisomi

**Affiliations:** 1 Division of Epidemiology and Biostatistics, School of Public Health, Faculty of Health Sciences, University of the Witwatersrand, Johannesburg, South Africa; 2 Department of Community and Environmental Health, School of Global and Public Health, Faculty of Health Sciences, Kamuzu University of Health Sciences, Blantyre, Malawi; 3 Department of Pathology, Kamuzu University of Health Sciences, Blantyre, Malawi; 4 School of Public Health, Faculty of Health Sciences, University of the Witwatersrand, Johannesburg, South Africa; 5 Department of Monitoring and Evaluation, Nigerian Institute of Medical Research, Lagos, Nigeria

**Keywords:** tuberculosis, child contacts, isoniazid preventive therapy, completion of IPT, adherence

## Abstract

**Background:**

The World Health Organization recommends 6 months of isoniazid preventive therapy (IPT) to children who have been exposed to tuberculosis (TB) patients to prevent active TB. Although IPT is an efficacious intervention, it is underutilised.

**Aim:**

The aim of this study was to examine factors associated with the completion of IPT among children under 5 years who have been exposed to TB patients.

**Setting:**

This was a secondary data analysis; the primary study was conducted in Blantyre, Malawi.

**Methods:**

This study was a secondary analysis of a randomised controlled trial with follow-up at 3 months and 6 months. Univariable and multivariable logistic regression models were used to identify factors associated with the completion of IPT.

**Results:**

One hundred and twenty-eight children were included, of whom 58 (45.3%) completed IPT. Index patient human immunodeficiency virus (HIV)-positive status (adjusted odds ratio [aOR] = 0.39, 95% confidence interval [CI]: 0.16–0.94) and longer distance (> 5 km) (aOR = 0.25, 95% CI: 0.07–0.89) were associated with lower IPT completion. Wealth status, household health-seeking decision maker and type of contact tracing were associated with higher IPT completion, with aOR = 3.42 (95% CI: 1.19–9.88) for children coming from households of high wealth status, aOR = 3.17 (95% CI: 1.19–8.42) in which the health-seeking decision maker was the parent compared to other guardians, and aOR = 3.13 (95% CI: 1.25–7.84) for children who were identified through patient-conducted tracing compared to routine contact tracing.

**Conclusion:**

Human immunodeficiency virus status, wealth status, household health-seeking decision maker, proximity to health facility and type of contact tracing are key determinants of IPT completion among children.

**Contribution:**

This study provides valuable insights into the factors that affect the completion of IPT. By addressing these factors, completion of IPT can be improved, thereby preventing TB among children.

## Introduction

Tuberculosis (TB) is one of the leading causes of morbidity and mortality globally and remains a public health concern.^1^ One of the most vulnerable groups affected by TB is children. Children who live with TB patients have a higher risk of getting infected with the disease as a result of their weak immune systems.^2,3^ According to the World Health Organization (WHO) global estimates, nearly 10 million people have TB annually, of which 1.2 million are children. Furthermore, every year, 1.5 million deaths are attributed to TB, of which 14% are among children.^1,3^ Malawi is among the TB-endemic countries in Africa. In 2022, Malawi had an estimated TB incidence of 125 per 100 000 population. In the same year, 18 000 people developed TB in Malawi, which included 2800 children.^3,4^

In response to the burden of TB, the WHO recommends tuberculosis preventive therapy (TPT) such as isoniazid preventive therapy (IPT) among child contacts of TB patients, which is to be taken once daily for a period of 6 months as one way of preventing progression to active TB.^5,6^ In addition to IPT, the WHO also recommends shorter TPT regimens such as rifapentine, also known as 3 months of isoniazid (H) and rifapentine (P) (3HP), which is to be taken once weekly for a period of 3 months and 1HP, which is to be taken once daily for a period of 28 days.^5^ Tuberculosis preventive therapy is also in line with achieving the WHO End TB strategy target, which aims to reduce the incidence of TB by 90% by 2035. Tuberculosis preventive therapy is also one of the top 10 indicators for monitoring the End TB Strategy’s performance.^7^ The effectiveness of IPT greatly depends on adherence and completion.^5^ However, despite the development of the WHO guidelines for administering IPT to child contacts, the effective implementation of these guidelines has not been optimal, especially in countries with high TB burden, including Malawi.^8^ Thus, the uptake and completion of IPT remain low.^8^ Isoniazid preventive therapy is inconsistently given to child contacts, and a gap exists between the expected number of child contacts eligible for screening and those actually starting and completing IPT.^9^

A report by the WHO in 2018 estimated that out of 1.3 million child contacts that were eligible for IPT, only 27% commenced IPT.^1,3^ According to the Malawi National TB control programme 2020 annual report, there were 9835 new confirmed TB index cases with 5025 estimated child contacts who were eligible for IPT. However, only 2364 (47%) of the child contacts completed IPT.^4^ In Malawi, the programmatic context has not significantly evolved in the period from 2014 to 2020. The completion of IPT among eligible child contacts remains low (35% in 2014, 40% in 2016, 46% in 2018 and 47% in 2020).^4^ This finding suggests the existence of barriers to completion of IPT among child contacts in Malawi.^4^ Existing literature highlights several hindrances to completion of IPT across different countries, including presence of side effects of IPT,^6,10,11,12^ duration of IPT,^13^ pill burden,^6,14^ drug stockouts,^11,15^ age of the child,^16^ sex of the child,^9,17^ type of TB of the index case,^18^ HIV status of the index case,^2,9,12^ level of education of the household head and/or caregiver,^2,19,20^ socioeconomic status of the household,^9,20,21^ number of under-five children in the household^12^ and distance to the health facility.^9,21^ However, the factors that are associated with the completion of IPT among child contacts in Malawi remain unclear, as there is a dearth of published evidence on these factors in Malawi. Thus, this study examined the factors associated with the completion of 6 months of IPT among children under 5 years, who have been exposed to TB patients in Blantyre, Malawi.^3^ Identification of these factors will aid in developing strategies and targeted interventions that will improve IPT completion in the population, thereby reducing TB incidence among child contacts in Malawi and contributing to the achievement of the WHO End TB Strategy.

## Research methods and design

### Study design

This study was a secondary analysis of a randomised controlled trial (RCT) titled ‘Sustainable household contact tracing and screening for tuberculosis patients and families’. The RCT (Trial registration number: ISRCTN81659509) aimed to implement and assess the effectiveness of a home-based TB screening strategy delivered at TB patients’ households. In the RCT, the study participants were randomly assigned to^1^ a standard of care group that followed standard and/or routine household screening, in which TB index patients were told to bring their household contacts for TB screening to the facility or^2^ the intervention group, called patient-delivered household active case finding for TB (PACTS), which involved the home-based TB screening strategy. Participants assigned to the intervention group were given a screening and/or triage tool and sputum collection pots for each household contact that was reported. The screening tool comprised information such as symptom screening, IPT eligibility for household contacts and sputum collection for the contacts. This screening was carried out by either the index patient or the parent and/or caregiver in the household. The TB screening was performed for both adult and child contacts. All child contacts were followed up at 3 months and 6 months for uptake and completion of IPT.^22^ Isoniazid preventive therapy completion was ascertained through clinical records, caregiver reports and inspection of medication bottles at 3- and 6-month visits. The study was conducted from May 2014 to November 2015 at Queen Elizabeth Central Hospital (QECH) in Blantyre, Malawi. The QECH is one of the tertiary-level facilities and one of the largest hospitals in Malawi. Most TB cases are diagnosed and managed at this hospital.

### Study population

The study population for this study was all under-five child contacts identified from TB cases in the RCT, irrespective of the trial arm in which the index case was in the RCT. These children were 128 in total.

### Variables used in the study

The dependent variable of this study was the completion of 6 months of IPT among all child contacts. The treatment outcome variable was coded as binary ‘IPT completed’ and ‘IPT not completed’.

The independent variables were at various levels. Contact characteristics included age, sex and relationship with the household head. Variables assessed also included index patient characteristics such as index case TB type and human immunodeficiency virus (HIV) status, which were used in the same way that they were originally coded in the primary study. Household characteristics such as household head employment, household head education level, health-seeking decision maker and wealth quintile consisted of the following categories in the primary study: least poor, less poor than average, average, poorer than average and poorest. These were re-categorised into three categories: high wealth status, medium wealth status and low-wealth status. Thus, ‘least poor’ and ‘less than poor than average’ were grouped as high wealth status, average was grouped as medium wealth status, and ‘poorer than average’ and ‘poorest’ were grouped as low-wealth status. For health access characteristics, the variables used were distance to the health facility (in kilometres) and the type of contact tracing and screening used. For distance, in the primary study, the distance variable had four categories, namely, ‘less than 1 km’, ‘more than 1 km but less than 5 km’, ‘more than 5 km but less than 10 km’ and ‘more than 10 km’. For the purpose of this study, the variable was categorised into two categories: ‘5 km or less’ and ‘more than 5 km’. The variable type of contact tracing and screening used a binary variable (‘patient-conducted tracing’ and ‘routine contact tracing’).

### Data analysis

A descriptive analysis of the distribution of baseline characteristics was performed. For categorical variables, percentages and frequencies were reported. Numerical variables were summarised as means and standard deviations if normally distributed and as median and interquartile range if not normally distributed. The prevalence of completion of IPT was estimated across all explanatory variables. This prevalence was reported as percentages for categorical variables, and *t*-test and Wilcoxon rank sum test were used for numerical variables in which medians or interquartile ranges (IQR) were compared. Binary logistic regression models were used to investigate factors associated with completion of IPT. Univariable and multivariable analyses were conducted. Unadjusted and adjusted odds ratios and 95% confidence intervals (CI) were reported. For building the multivariable regression model, stepwise regression was used: sex, age of the child and index HIV status were included a priori, and other variables were included if found significant from the univariable logistic regression model. Variables with *p*-values < 0.20 in univariable analysis or those supported by literature were included in the multivariable model. For handling of missing data, we used complete case analysis.

### Data source

PACTS trial data were used (data from the primary study) in this study. Permission to use the data was sought and was approved by the principal investigator of the primary study, Dr Kruger Kaswaswa from the Kamuzu University of Health Sciences (KUHES). Data were obtained from the principal investigator of the primary study.

### Ethical considerations

Ethical clearance to conduct this study was obtained from the University of the Witwatersrand Human Research Ethics Committee (No. M230121 MED22-10-172). The primary study was approved by the Institutional Review Board for Dr Kruger Kaswaswa from the Kamuzu University of Health Sciences (KUHES) in Malawi. The primary participants provided consent for the primary study, including the use of anonymised data for secondary data analyses. The data shared were anonymised, and there was no identifying information. This study was a secondary data analysis; thus, the ethics review did not require individual informed consents.

## Results

**FIGURE 1:**
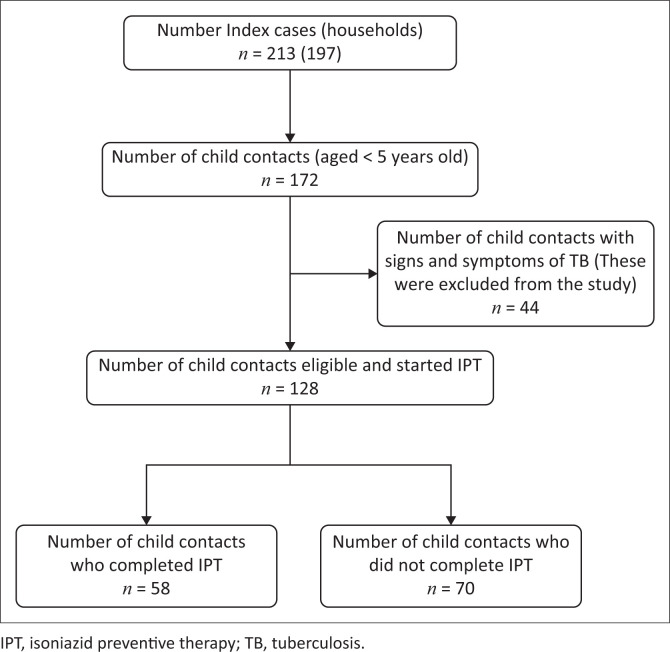
Flowchart showing the number of children included in the study.

Figure 1 is a flow diagram of child contacts included in the study. According to the primary study, 213 index patients were identified from 197 households. There were 172 child contacts under 5 years, who were identified from the 213 index cases, of which 128 were eligible and referred for initiation of IPT. Forty-four child contacts were not eligible to start IPT because they had signs and symptoms of TB during TB screening and assessment. Therefore, this study had 128 child contacts who had IPT completion status assessed.

### Baseline characteristics

Table 1 shows the baseline characteristics of the child contacts, index case characteristics, household characteristics and health access characteristics. The female child contacts constituted 52.0% of the study population. The median age of the child contacts was 3 (IQR: 1–4), and 71.9% were children of the household head. About two-thirds of the related-index cases had smear-positive TB, and 52.3% were HIV-positive. About half of the child contacts came from households with low-wealth class (52.3%), 73.4% of their household heads were employed, and 53.9% had no school and/or primary level of education. The median number of children under five in the households was two (IQR: 1–3). The majority of the child contacts had their parents (68.8%) as the main healthcare-seeking decision maker, and the median number of people living in households was six (IQR: 4–8). Over four to five (82.0%) of the children’s contacts lived at a distance of 5 km or less from the health facility, and 51.6% were identified through routine contact tracing.

**TABLE 1:** Description of child contact characteristics, index case characteristics, household characteristics and health access characteristics of the child contacts.

Variables	Variable categories	*n*	%	Median	IQR
Total	-	128	-	-	-
**Child contact characteristics**	-	-	-	-	-
Sex	Female	66	52.0	-	-
	Male	62	48.0	-	-
Age in years	-	-	-	3	1–4
Relationship with household head	Other	36	28.1	-	-
	Child	92	71.9	-	-
**Index case characteristics**	-	-	-	-	-
TB type	Smear-negative TB	43	33.6	-	-
	Smear-positive TB	85	66.4	-	-
HIV status of the index case†	HIV-negative	50	39.1	-	-
	HIV-positive	67	52.3	-	-
**Household characteristics**	-	-	-	-	-
Wealth status‡	Low	67	52.3	-	-
	Medium	22	17.2	-	-
	High	37	28.9	-	-
Household head’s employment status	Unemployed	34	26.6	-	-
	Employed	94	73.4	-	-
Household head’s education level	No school and/or primary	69	53.9	-	-
	Secondary and/or higher	59	46.1	-	-
Number of < 5 children in the household	-	-	-	2	1–3
Household health-seeking decision maker	Other guardians	40	31.2	-	-
	Parents	88	68.8	-	-
Number of people living in the household	-	-	-	6	4–8
**Health access characteristics**	-	-	-	-	-
Distance to health facility (km)	5 km or less	105	82.0	-	-
	More than 5 km	23	18.0	-	-
Type of contact tracing	Routine contact tracing	66	51.6	-	-
	Patient-conducted tracing	62	48.4	-	-

HIV, human immunodeficiency virus; TB, tuberculosis; IQR, interquartile range.

† , There were 11 index cases with unknown HIV status;

‡ , There were two child contacts with missing information on wealth status.

### Prevalence of isoniazid preventive therapy completion among child contacts by different characteristics

Table 2 presents IPT completion among child contacts by different characteristics. Overall, 45.3% of the child contacts completed IPT. Completion of IPT was high among female child contacts (48.5%) compared to male child contacts (41.9%), but this difference was insignificant (*p* = 0.46). The child contacts that were younger had a slightly higher completion rate with a median age of 2 (IQR: 1–3), even though it was not statistically significant (*p* = 0.26). Child contacts who had the head of the household as their parent had a higher completion of 47.8% compared to that for child contacts who were not children of the household head (38.9%) (*p* = 0.36).

**TABLE 2:** Completion of isoniazid preventive therapy among child contacts by different characteristics.

Variables	Variable categories	Total (*n*)	IPT completed				IPT not completed				*p-*value**
			*n*	%	Median	IQR	*n*	%	Median	IQR	
Total	-	128	58	-	-	-	70	-	-	-	-
**Child contact characteristics**	-	-	-	-	-	-	-	-	-	-	-
Sex	Female	66	32	48.5	-	-	34	51.5	-	-	0.46
	Male	62	26	41.9	-	-	36	58.1	-	-	-
Age in years	-	128	-	-	2	1–3	-	-	3	1–4	0.26
Relationship with household head	Other	36	14	38.9	-	-	22	61.1	-	-	0.36
	Child	92	44	47.8	-	-	48	52.2	-	-	-
**Index case characteristics**	-	-	-	-	-	-	-	-	-	-	-
TB type	Smear-negative TB	43	20	46.5	-	-	23	53.5	-	-	0.85
	Smear-positive TB	85	38	47.7	-	-	47	55.3	-	-	-
HIV status of the index case†	HIV-negative	50	28	56.0	-	-	22	44.0	-	-	0.07
	HIV-positive	67	26	38.8	-	-	41	61.2	-	-	-
**Household characteristics**	-	-	-	-	-	-	-	-	-	-	-
Wealth status‡	Low	67	23	34.3	-	-	44	65.7	-	-	0.01
	Medium	22	10	45.5	-	-	12	54.5	-	-	-
	High	37	24	64.9	-	-	13	35.1	-	-	-
Household head’s employment status	Unemployed	34	14	41.2	-	-	20	58.8	-	-	0.57
	Employed	94	44	46.8	-	-	50	53.2	-	-	-
Household head’s education level	No school and/or primary	69	28	40.6	-	-	41	59.4	-	-	0.25
	Secondary and/or higher	59	30	50.8	-	-	29	49.2	-	-	
Number of < 5 children in the household	-	128	-	-	2	1–3	-	-	2	1–3	0.15
Household health-seeking	Other guardians	40	11	27.5	-	-	29	72.5	-	-	0.01
Decision maker	Parents	88	47	53.4	-	-	41	46.6	-	-	-
Number of people living in the household	-	128	-	-	5	4–6	-	-	6	5–8	0.01
**Health access characteristics**	-	-	-	-	-	-	-	-	-	-	-
Distance to health facility (km)	5 km or less	105	52	49.5	-	-	53	50.5	-	-	0.04
	More than 5 km	23	6	26.1	-	-	17	73.9	-	-	-
Type of contact tracing	Routine contact tracing	66	25	37.9	-	-	41	62.1	-	-	0.08
	Patient-conducted tracing	62	33	53.2	-	-	29	46.8	-	-	-

IPT, isoniazid preventive therapy; IQR, interquartile range; TB, tuberculosis; HIV, human immunodeficiency virus.

† , There were 11 index cases with unknown HIV status;

‡ , There were 2 child contacts with missing information on wealth status.

Isoniazid preventive therapy completion was slightly higher for child contacts whose related-index case had smear-negative TB type (46.5%) than (44.7%) for the smear-positive TB (*p* = 0.85). Isoniazid preventive therapy completion was high among child contacts whose index case was HIV-negative (56.0%) (*p* = 0.07) compared to that of child contacts whose index case was HIV-positive (38.8%).

Children from high-wealth status households had a significantly higher completion of 64.9% compared to that of children from low-wealth status (34.3%) (*p* = 0.01). Completion was slightly higher in households in which the household head was employed (46.8%) and had a secondary and/or higher level of education (50.8%). The median number of under-five children in the household who completed IPT was two (IQR: 1–3). Completion was higher among child contacts whose parent was the health-seeking decision maker (53.4%), compared to other relatives (27.5%) (*p* = 0.01). The median number of people living in the household who completed IPT was five (IQR: 4–6) (*p* = 0.01).

Child contacts that lived at a distance of 5 km or less from the health facility had significantly higher prevalence of IPT completion (49.5%), compared to those that lived further away (26.1%) (*p* = 0.04). There was higher completion among child contacts who were identified through the patient-conducted tracing (53.2%) compared to those who were identified through routine contact tracing (37.9%) (*p* = 0.08).

### Factors associated with completion of isoniazid preventive therapy

Table 3 shows the univariable and multivariable analyses for the completion of IPT. For wealth status, the odds of completing IPT among child contacts who came from high wealth status were 4.18 times the odds of completing IPT among child contacts who came from low-wealth status (odds ratio [OR] = 4.18, 95% CI: 1.70–10.25, *p* = 0.01). The role of the household healthcare decision maker affected IPT completion; the odds of completing IPT among child contacts whose parents were the household health-seeking decision maker were 2.58 times the odds of completing IPT among child contacts whose other guardian was the household health-seeking decision maker (OR = 2.58, 95% CI: 1.12–5.95, *p* = 0.02). For the number of people living in the household, the odds of completing IPT were 0.81 (OR = 0.81, 95% CI: 0.69–0.96, *p* = 0.01) for each unit increase in the number of people in the household.

**TABLE 3:** Factors associated with the completion of isoniazid preventive therapy among child contacts.

Variables	Variable categories	Univariable		Multivariable	
		OR	95% CI	OR	95% CI
**Child contact characteristics**	-	-	-	-	-
Sex	Female	1.00	-	1.00	-
	Male	0.89	0.43–1.86	0.86	0.36–2.06
Age in years	-	0.91	0.69–1.20	0.89	0.63–1.26
Relationship with household head	Other	1.00	-	-	-
	Child	1.69	0.72–3.97	-	-
**Index case characteristics**	-	-	-	-	-
TB type	Smear-negative TB	1.00	-	-	-
	Smear-positive TB	0.86	0.39–1.88	-	-
HIV status of the index case	HIV-negative	1.00	-	1.00	-
	HIV-positive	0.49	0.23–1.04	0.39	0.16–0.94*
**Household characteristics**	-	-	-	-	-
Wealth status	Low	1.00	-	1.00	-
	Medium	1.82	0.66–5.00	1.11	0.31–3.93
	High	4.18	1.70–10.25*	3.42	1.19–9.88*
Household head’s employment status	Unemployed	1.00	-	-	-
	Employed	1.47	0.64–3.33	-	-
Household head’s education level	No school and/or primary	1.00	-	-	-
	Secondary and/or higher	1.57	0.75–3.28	-	-
Number of < 5 children in the household	-	0.64	0.39–1.07	-	-
Household health-seeking decision	Other guardians	1.00	-	1.00	-
Maker	Parents	2.58	1.12–5.95*	3.17	1.19–8.42*
Number of people living in the household	-	0.81	0.69–0.96*	0.87	0.71–1.06
**Health access characteristics**	-	-	-	-	-
Distance to health facility (km)	5 km or less	1.00	-	1.00	-
	More than 5 km	0.30	0.10–0.88*	0.25	0.07–0.89*
Type of contact tracing	Routine contact tracing	1.00	-	1.00	-
	Patient-conducted tracing	2.41	1.14–5.11*	3.13	1.25–7.84*

Note: The number of children was 115 from 82 households. In the model, adjusting for clustering household or index case variables was non-significant, for example, index HIV status, wealth status, and household health-seeking decision maker. The variables significant after adjusting for clustering were facility distance > 5 km, aOR: 0.25 (95% CI: 0.07–0.90) and contact tracing method by patient-delivered tracing, aOR 3.12 (1.17–8.34).

OR, odds ratio; HIV, human immunodeficiency virus; TB, tuberculosis.

* , *p*-value < 0.05.

Distance to a health facility was also important. The odds of completing IPT among child contacts whose distance to the health facility was more than 5 km were 0.30 times the odds of completing IPT among child contacts whose distance to the health facility was < 5 km (OR = 0.30, 95% CI: 0.10–0.88, *p* = 0.05). For the type of contact tracing, the odds of completing IPT among child contacts who were identified through patient-conducted tracing were 2.41 times the odds of completing IPT among child contacts who were identified through routine contact tracing (OR = 2.41, 95% CI: 1.14–5.11, *p* = 0.02).

On multivariable analysis, wealth status was significantly associated with IPT completion. Higher wealth status was significantly associated with higher completion of IPT among children, in whom the adjusted OR (aOR) for IPT completion was 3.42 (95% CI: 1.19–9.88, *p* = 0.02) for children coming from households of high wealth status, compared to those coming from the lowest wealth status. A significant association was noted after multivariate analysis between the HIV status of the child contacts’ index case and the completion of IPT; child contacts with HIV-positive index cases had significantly lower odds of IPT completion, with aOR = 0.39 (95% CI: 0.16–0.94, *p* = 0.02). Household health-seeking decision maker was still significantly associated with completion of IPT, with aOR = 3.17 (95% CI: 1.19–8.42, *p* = 0.03) for child contacts whose parents were the household health-seeking decision maker, compared to child contacts who had other guardians as the household healthcare decision maker.

After the multivariable analysis, the number of people living in the household was not significantly associated with completion of IPT, with aOR = 0.87 (95% CI: 0.71–1.06, *p* = 0.61) for each unit increase in the number of people living in the household. The distance to a health facility was also still significantly associated with IPT completion, with aOR = 0.25 (95% CI: 0.07–0.89, *p* = 0.03) for child contacts living at a distance of more than 5 km, compared to those at a distance of less than 5 km. Similarly, the type of contact tracing was still significantly associated with the completion of IPT, with aOR of 3.13 (95% CI: 1.25–7.84, *p* = 0.01) for child contacts who were identified through patient-conducted tracing, compared to child contacts who were identified through routine contact tracing.

## Discussion

This study investigated the factors associated with the completion of 6 months of IPT among children under 5 years, exposed to TB patients in Blantyre, Malawi. This study found that a longer distance to the health facility (> 5 km) was significantly associated with lower completion of IPT. This observation is coherent with other previous studies that have also found a significant association between the distance to the health facility and completion of IPT and have highlighted the influence of healthcare accessibility on treatment adherence.^16,17^ Distance to the health facility also relates to transportation barriers, as health facilities that are far pose a major hindrance to completion of IPT.^17^ Lack of reliable transportation options, especially in rural areas, can hinder regular visits to the health facility, which may lead to missed doses of IPT.^17^

This study found a significantly higher rate of completion of IPT among child contacts who had their parents as the household’s healthcare-seeking decision maker as opposed to other guardians. This finding highlights the importance of understanding the role of the decision maker in the utilisation of healthcare services in the household and treatment adherence.^3^ This study finding aligns with a previous study that highlighted how the decision-making authority in a household significantly influences health-seeking behaviours and treatment completion.^23^ Health-seeking behaviour for TB services is even better when parents have better knowledge about TB. It is important to note that the household health-seeking decision maker’s knowledge about TB and the importance of IPT may influence and motivate them to seek healthcare for the child contacts and ensure that they complete IPT.^23^

This study found that child contacts whose index case was HIV-positive were less likely to complete IPT, compared to child contacts whose index cases were HIV-negative.^3^ This finding replicates the finding of a study conducted in Rwanda, which also found the same result.^24^ This result relates to the fact that often, HIV-positive people have to cope with social stigma, which affects their health-seeking behaviour.^24^

Household wealth status is important in determining the access to health services of the people in that household. In this study, higher wealth status was associated with higher IPT completion. This study finding is in alignment with an analytical cross-sectional study that found a significant association between household wealth status and completion of IPT.^16^ However, a mixed-method study conducted in Rwanda found no significant association between wealth status and completion of IPT.^9^ The different study designs used in the different studies could be a reason for the contradictory findings. In addition, the measurement and definition of wealth status across studies may have led to the variations in the findings.^9,16^

This study found that the type of contact tracing was significantly associated with the completion of IPT among child contacts. That is to say, children from households in which the index TB patients and/or guardians were trained to conduct household screening and encourage IPT initiation (intervention of primary study) had a higher rate of completion compared to the standard of care (routine contact tracing).^3^ This finding is in accordance with the findings of an intervention study, which also found a significant association between the type of contact tracing and completion of IPT.^10^ Oftentimes, interventions to improve contact tracing improve IPT completion rates. Hence, interventions to improve contact tracing provide opportunities to mitigate the risk of TB among child contacts.^10,22,25^ The findings of this study highlight and emphasise the importance and the role of targeted efforts in actively identifying and engaging child contacts, which may enhance their access to IPT and improve treatment adherence and completion.

This study found that sociodemographic characteristics of the child contacts, such as their age and sex, were not significantly associated with the completion of IPT. Similar to this finding, three studies conducted in Nigeria, Guinea-Bissau and Ethiopia also found no significant association between the age and sex of the child contact and the completion of IPT,^12,26,27^ meaning that the sex and age of the child contact did not influence the completion of IPT. In addition, sex and age of the child contacts alone were not strong predictors and/or significant determinants of IPT completion, suggesting that there may be other factors that may play a more significant role in predicting IPT completion.^27^

The associations observed in this study may be explained by several plausible mechanisms. Shorter distances to health facilities may be linked to lower transport costs and less travel time, thereby contributing to adherence to health facility visits and IPT completion.^17^ Completion was higher when the primary healthcare decision maker was the parent. This result may reflect the health literacy of the parents and the authority they have in their household and their commitment to ensuring that their children complete IPT.^23^ High wealth status may ease financial barriers such as transport costs to the health facility and consistent adherence to health facility visits.^16^ The use of patient-conducted contact tracing brought TB screening to the household, which reduced health access barriers and promoted easy access to IPT adherence support.^10^ Lower IPT completion among child contacts whose index case was HIV-positive may be linked to the stigma associated with HIV status and competing demands related to frequent health facility visits for HIV care.^24^ These pathways are plausible explanations but were not directly measured in this study.

The primary study was conducted from 2014 to 2015, when the main TPT option used in Malawi was IPT. Since then, shorter regimens such as 3HP and 1HP have been introduced and are recommended; however, the factors affecting IPT completion such as distance to health facility, wealth status, healthcare decision maker and type of contact tracing that have been identified in this study, remain relevant, and they still inform access and adherence barriers even for the shorter regimens that have been introduced.

### Limitations

Although this study provides valuable insights into factors associated with the completion of IPT, the study had some limitations. The primary study had information on uptake and completion of IPT available, but information on actual drug adherence (such as pill count) was not available, which may have resulted in an overestimation of the number of participants who received an effective course of IPT and completed the whole course of IPT. Literature has also found that other relevant factors associated with completion of IPT, such as duration of IPT, presence of side effects, pill burden, pill size, pill taste, health factors (drug stock outs) and direct observed therapy; however, these were not included in this study, because the dataset did not have the variables. In addition, this study used a small sample size, which may have increased the risk of model overfitting. We did not account for household clustering in the model, which could have introduced bias if outcomes were correlated among children from the same household. There is potential for residual confounding given the observational nature of the data for some variables, such as distance to the health facility (if self-reported) and the healthcare-seeking decision maker, which may be subject to measurement error. Furthermore, the use of a complete case analysis to handle missing data may have introduced bias.

### Recommendations

Our findings suggest that IPT completion among child contacts is shaped by both health facility access and household factors. Distance to the clinic highlights the need to strengthen peripheral delivery or provide transport support. Especially in rural areas, this can be achieved through community health posts, mobile clinics and outreach clinics. We recommend the need for counselling, awareness, family-centred engagement and/or support and health education for guardians other than parents who make healthcare decisions in the households. This will assist in ensuring that the guardians are empowered and are fully informed about IPT and the importance of IPT completion, thus facilitating the care for the child contacts. Closer integration of IPT with HIV services and offering of targeted adherence support may help to mitigate the challenge of stigma and balance competing health demands when the index case is HIV-positive, thereby improving IPT completion. Supporting patient contact tracing and exploring support targeted for low-wealth status households could also improve IPT uptake and completion. Future studies should investigate other factors that may affect the completion of IPT, for instance, how household dynamics and different community-based delivery models affect IPT completion. This effort will guide more effective policies and practices.

## Conclusion

In conclusion, this research investigated the factors associated with the completion of IPT among child contacts. Isoniazid preventive therapy is one of the effective ways of preventing TB among child contacts; the effectiveness of IPT greatly depends on completion. Therefore, it is important to understand the factors that affect the completion of IPT among child contacts. This study has found that completion of IPT is suboptimal, which needs to be improved. The findings of this study highlighted several factors that are associated with the completion of IPT: HIV status of the index case, wealth status, household healthcare decision maker, distance to the health facility and the type of contact tracing.

The findings of this study demonstrate the contribution of implementation research to health programmes. In addition, this study provides valuable insights into the factors that affect the completion of IPT. By addressing the factors identified, completion of IPT can be improved, thereby preventing TB among children, which is in line with the WHO End TB 2030 strategy. Therefore, there is a need to find approaches that address and enhance IPT completion.
